# Comparative Wear of Opposing Natural Enamel by Different Ceramic Materials in Fixed Dental Protheses: A Systematic Review and Meta-Analysis

**DOI:** 10.3390/dj14010037

**Published:** 2026-01-06

**Authors:** Cleber Davi Del Rei Daltro Rosa, Victor Augusto Alves Bento, Nathália Dantas Duarte, Jéssica Marcela de Luna Gomes, Roberta Okamoto, Rogerio Leone Buchaim, Daniela Vieira Buchaim, João Paulo Mardegan Issa, Eduardo Piza Pellizzer

**Affiliations:** 1 Department of Dental Materials and Prosthodontics, School of Dentistry, São Paulo State University (FOA-UNESP), Araçatuba 16015-050, Brazil; cleber.davi@unesp.br (C.D.D.R.D.R.); jessica.gomes@unesp.br (J.M.d.L.G.); 2 Department of Prosthodontics, School of Dentistry, Federal University of Mato Grosso do Sul (UFMS), Campo Grande 79070-140, Brazil; augusto.alves@ufms.br; 3 Department of Diagnosis and Surgery, School of Dentistry, São Paulo State University (FOA-UNESP), Araçatuba 16015-050, Brazil; nd.duarte@unesp.br; 4 Department of Basic Sciences, School of Dentistry, São Paulo State University (FOA-UNESP), Araçatuba 16015-050, Brazil; roberta.okamoto@unesp.br; 5 Graduate Program in Anatomy of Domestic and Wild Animals, Faculty of Veterinary Medicine and Animal Science, University of São Paulo (FMVZ-USP), São Paulo 05508-270, Brazil; rogerio@fob.usp.br (R.L.B.); danibuchaim@alumni.usp.br (D.V.B.); 6 Department of Biological Sciences, School of Dentistry, University of São Paulo (FOB-USP), Bauru 17012-901, Brazil; 7 Department of Anatomy, School of Medicine, University Center of Adamantina (FAI), Adamantina 17800-000, Brazil; 8 Department of Postgraduate, School of Dentistry, Faculty of Midwest Paulista (FACOP), Piratininga 17499-010, Brazil; 9 Department of Basic and Oral Biology, School of Dentistry, University of São Paulo (FORP USP), Ribeirão Preto 14040-904, Brazil; jpmissa@forp.usp.br

**Keywords:** ceramic materials, crowns, dental wear, enamel, systematic review

## Abstract

**Background/Objectives**: This systematic review aimed to quantify the extent of wear of opposing posterior natural enamel in patients with single-unit ceramic crowns. **Methods**: A comprehensive search was conducted in PubMed/MEDLINE, Embase, Web of Science, Cochrane Library, and ProQuest through September 2025. A meta-analysis was performed using the inverse variance method. **Results**: Nine clinical studies (5 randomized controlled trials and 4 prospective studies) involving 203 patients (2015–2025) were included. All studies evaluated monolithic zirconia; two also assessed monolithic lithium disilicate, and three included metal-ceramic restorations with feldspathic veneering. Follow-up ranged from 6 to 24 months. Meta-analysis revealed significant enamel wear from zirconia (*p* < 0.05; MD: −1.32; 95% CI: −2.06 to −0.57; I^2^ = 94%) and lithium disilicate (*p* < 0.05; MD: −0.45; 95% CI: −0.71 to −0.19; I^2^ = 2%). Feldspathic ceramics did not show significant enamel wear (*p* = 0.06; MD: −2.77; 95% CI: −5.66 to 0.13; I^2^ = 96%). **Conclusions**: Ceramic materials generally cause greater wear on opposing posterior natural enamel than enamel-to-enamel contact. Monolithic zirconia and lithium disilicate crowns produced higher antagonist wear, whereas metal-ceramic restorations with feldspathic veneering appeared more conservative for preserving posterior enamel.

## 1. Introduction

Tooth wear is a multifactorial loss resulting from direct contact between dental enamel and restorations. This phenomenon may result from physiological or pathological causes, in which biological, mechanical, or chemical factors may play a determining role in the progression of tooth wear, thereby classifying it as erosion, abrasion, or attrition [1]. Approximately 30–40% of patients rehabilitated with fixed prostheses exhibit some degree of antagonist enamel wear over time [1,2]. This process may lead to a reduction in the vertical dimension of occlusion, alterations in masticatory efficiency, and even functional discomfort [2].

Selecting an appropriate ceramic material is crucial for minimizing these adverse effects and ensuring a balance between mechanical strength and compatibility with natural enamel. Ideally, the wear of ceramic materials should be similar to that of natural enamel. However, the complexity and progression of tooth wear pose challenges to the development of reliable studies [3]. Properties such as toughness, fracture resistance, surface roughness, and hardness have been identified as key contributors to increased tooth wear [4,5].

The average annual enamel wear between opposing natural teeth ranges from 20 to 40 µm, whereas enamel wear against various ceramic materials shows wide variability [6,7,8,9,10]. Full ceramic restorations have become increasingly popular due to their metal-free composition, esthetics, and biocompatibility. Notably, those fabricated using CAD-CAM technology with tetragonal yttria-stabilized zirconia (3%, 4%, 5% Y-TZP) exhibit high hardness and fracture resistance, enabling minimal thicknesses of approximately 0.5 mm, thereby contributing to the biological benefit of preserving tooth structure [11,12].

This has led to the widespread adoption of monolithic zirconia (MZ) as an alternative to other ceramic types and traditional metal-ceramic restorations. Nevertheless, owing to its mechanical properties, this material type has the potential to increase wear of the opposing enamel [13]. Enamel wear can be evaluated using indirect or direct digital analysis. The indirect method employs laser scanning technology to create tooth replicas. However, technical factors, such as the laser beam wavelength, may significantly impact the accuracy of wear assessment [14].

Direct digital analysis, performed with intraoral scanners, enables reliable evaluation of enamel wear by eliminating errors associated with the replication process [14]. Although several systematic reviews of in vitro studies have been conducted, testing conditions across these studies have been inconsistent, making it impossible to simulate the complex conditions involved in tooth wear fully [15,16,17].

As a result, there is still no consensus on which ceramic material causes the higher enamel wear, and no systematic review with meta-analysis has quantitatively compared enamel wear across different ceramic materials in natural antagonist teeth over various follow-up periods. Therefore, this review aims to quantify which ceramic material causes the highest wear to the natural enamel of the posterior antagonist tooth in patients with single posterior crowns across different follow-up periods. The null hypothesis is that there will be no significant difference among the ceramic materials over time.

## 2. Materials and Methods

### 2.1. Protocol and Registration

This systematic review was conducted following the Preferred Reporting Items for Systematic Reviews and Meta-Analyses (PRISMA 2020). The PRISMA checklist is provided in Supplementary Materials (Table S1) [18]. A protocol was developed and registered in the International Prospective Register of Systematic Reviews (PROSPERO; registration number CRD42022327721).

### 2.2. Eligibility Criteria

Studies were included if they met the criteria: randomized clinical trials (RCTs) or prospective studies (non-RCTs). Studies may consist of at least 10 participants, quantitative measurements of antagonist tooth wear across different types of single-unit ceramic crowns, comparisons with natural antagonist posterior tooth wear, and long-term follow-up. The exclusion criteria included in vitro studies, case reports, studies without follow-up, and those lacking comparison with natural antagonist teeth (the control group).

### 2.3. PICO Question

The Population, Intervention, Comparison, Outcome (PICO) was “Which ceramic material (intervention) causes the highest wear (outcome) to the natural enamel of the posterior opposing tooth (comparison) in patients with single posterior ceramic crowns (population)?”

### 2.4. Search Strategy

Two members of the research team (C.D.D.R.D.R., V.A.A.B.) conducted electronic searches in PubMed/MEDLINE, Embase, Web of Science, and the Cochrane Library, covering studies published through September 2025, with no language or publication date restrictions. A tailored search strategy was created for each database. The complete set of search terms used is available in Table 1. No filters or database limits were applied. Additionally, manual searches were conducted in the reference lists of included articles and in gray literature using the ProQuest database (https://proquest.libguides.com/).

### 2.5. Study Selection Process

After conducting the systematic search, all identified citations were imported into the Rayyan QCRI reference manager (https://rayyan.ai/), accessed on 25 September 2025, and duplicates were removed. Titles and abstracts were screened by two independent reviewers (C.D.D.R.D.R.; V.A.A.B.) in accordance with the eligibility criteria. In cases of disagreement, a third reviewer (N.D.D.) was consulted, and consensus was reached.

### 2.6. Data Collection Process

One author (C.D.D.R.D.R.) extracted data from the included articles (qualitative or quantitative), and two authors (V.A.A.B.; N.D.D.) reviewed all collected information. The extracted variables for each study included author information, year, number of participants, mean age, sex, ceramic, single-unit crown location, wear measurement method, follow-up period, and results.

### 2.7. Quality Assessment

Two investigators (C.D.D.R.D.R.; V.A.A.B.) assessed the quality and risk of bias of RCTs using the RoB 2.0. This tool evaluates five specific domains: (1) bias arising from the randomization process; (2) bias due to deviations from intended interventions; (3) bias due to missing outcome data; (4) bias in outcome measurement; and (5) bias in the selection of reported results. Based on these domains, an overall bias rating was assigned to each study, categorized as low risk, high risk, or some concerns [19]. For non-randomized studies, the risk of bias was assessed using the ROBINS-I tool, which considers bias at the pre-intervention stage (bias due to confounding and selection of participants), intervention stage (bias in classification of interventions), and post-intervention stage (bias due to deviations from intended interventions, missing data, measurement of outcomes, and selection of reported results) [20].

### 2.8. Meta-Analysis

Two investigators (C.D.D.R.D.R.; V.A.A.B.) conducted the meta-analysis using the Inverse-Variance (IV) method. The wear values of enamel vs. enamel and enamel vs. ceramic were analyzed by calculating the standardized mean difference (SMD) of wear measurements in micrometers, as reported in the studies. All analyses were considered statistically significant at *p* < 0.05 with 95% confidence intervals (CI). When considerable heterogeneity was observed, a random-effects model was applied. Otherwise, a fixed-effects model was used. The Reviewer Manager Software (Version 5.4; Cochrane Group) was used.

### 2.9. Additional Analysis

An additional analysis was performed to evaluate inter-examiner agreement during the study selection process using the Kappa statistic. Any discrepancies were resolved through discussion and consensus among all authors.

## 3. Results

### 3.1. Search Strategy

**Figure 1:**
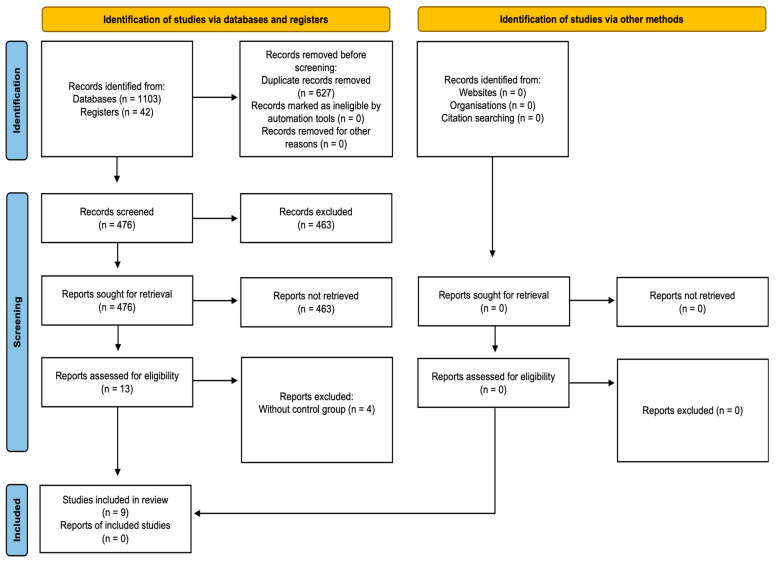
PRISMA flow diagram illustrating the search strategy details.

The initial electronic search identified 1103 articles: 385 from PubMed/MEDLINE, 378 from Embase, 278 from Web of Science, 42 from the Cochrane Library, and 20 from ProQuest and manual searching. A total of 627 duplicate references were removed, leaving 476 articles. After screening titles and abstracts in detail, 13 articles [14,21,22,23,24,25,26,27,28,29,30,31,32] met eligibility and exclusion criteria and were included in this systematic review. Of these, four articles [21,22,23,24] were excluded because they lacked a control group. The whole search strategy is outlined in Figure 1.

### 3.2. Characteristics of the Studies

A total of 9 clinical studies [14,25,26,27,28,29,30,31,32] were included in this systematic review for qualitative assessment, comprising 5 RCTs and 4 prospective studies, published between 2015 and 2025, and involving 203 patients. All studies evaluated vertical enamel wear (µm) of the antagonist tooth in contact with a single-unit crown. Each study included a group with monolithic zirconia ceramics; two included monolithic lithium disilicate ceramics; and three included metal-ceramic restorations with feldspathic ceramic veneer. Follow-up periods ranged from 6 to 24 months. The methods used to measure enamel wear varied across studies, including indirect replica-based laser scanning and direct intraoral scanning. These methodological differences and characteristics of the included studies are summarized in Table 2.

Polishing protocols were detailed in most of the studies and consistently emphasized the importance of surface finishing in reducing antagonist enamel wear. The majority of studies used sequential mechanical polishing with diamond abrasives, often followed by glaze application or diamond polishing pastes. For instance, Stober et al., 2016 [26] and Nazirkar et al., 2020 [27] highlighted the efficacy of combining diamond polishing with glazing in reducing roughness and, consequently, enamel wear. Esquivel-Upshaw et al., 2018 [11] employed a detailed, multi-step polishing procedure, culminating in the application of diamond paste with a stiff-bristled brush, which demonstrated favorable outcomes. Similarly, Selvaraj et al., 2021 [29] and Woraganjanaboon et al., 2024 [31] used structured polishing sequences with diamond instruments in various grit levels, achieving controlled surface roughness and reduced abrasiveness. Although Deval et al., 2021 [28] did not report specific polishing methods, the importance of adequate polishing to minimize antagonist wear was acknowledged. The characteristics of polishing techniques are included in Table 3.

### 3.3. Risk of Bias

**Figure 2:**
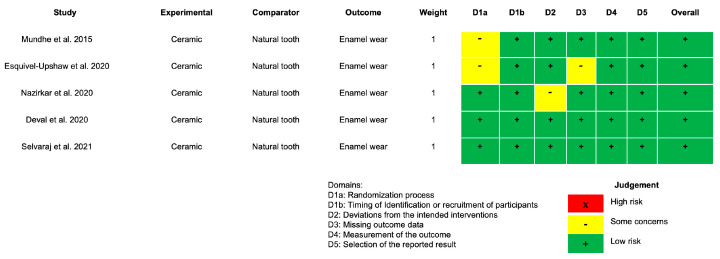
Risk of bias (RoB 2.0) for randomized clinical trials (RCTs) [14,25,27,28,29].

The assessment using the RoB 2.0 tool indicated unclear risk of bias in the studies by Mundhe et al., 2015 [25], Esquivel-Upshaw et al., 2020 [14], and Nazirkar et al., 2020 [27], particularly in domains D1, D2, and D3 (Figure 2). In the study by Mundhe et al., 2015 [25], the bias was attributed primarily to the lack of randomization and unclear recruitment procedures. In the study by Esquivel-Upshaw et al., 2020 [14], unclear bias was also observed, with a similar focus on the domains of patient selection (D1) and data collection (D3), as the details of recruitment and data collection methods were not clearly described. In the study by Nazirkar et al., 2020 [27], although it was approved by the Institutional Ethics Committee and adhered to informed consent guidelines, it was not formally registered in a clinical trial database.

**Figure 3:**
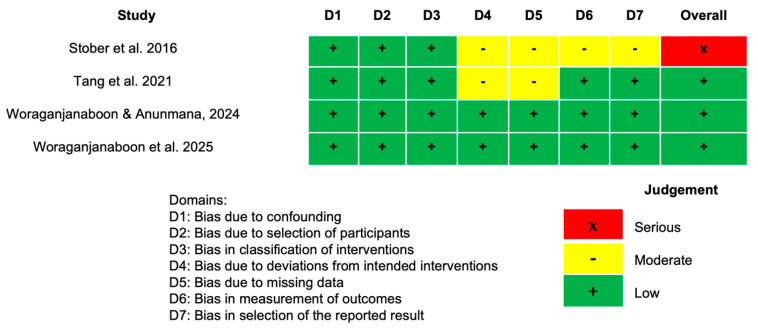
Risk of bias (ROBINS-I) for non-randomized clinical studies [26,30,31,32].

In the ROBINS-I tool, only the study by Stober et al., 2016 [26] was rated as high risk of bias across the domains of deviation from intended interventions, measurement and reporting of outcomes, and statistical analysis (D4, D5, D6, and D7), as shown in Figure 3. This risk was attributed to the lack of clarity regarding deviations from the intended interventions (D2). Furthermore, the measurement and reporting of outcomes were not sufficiently detailed, resulting in a high risk in domain D4. For statistical analysis, the lack of information about the study’s methods compromised transparency.

### 3.4. Meta-Analysis

**Figure 4:**
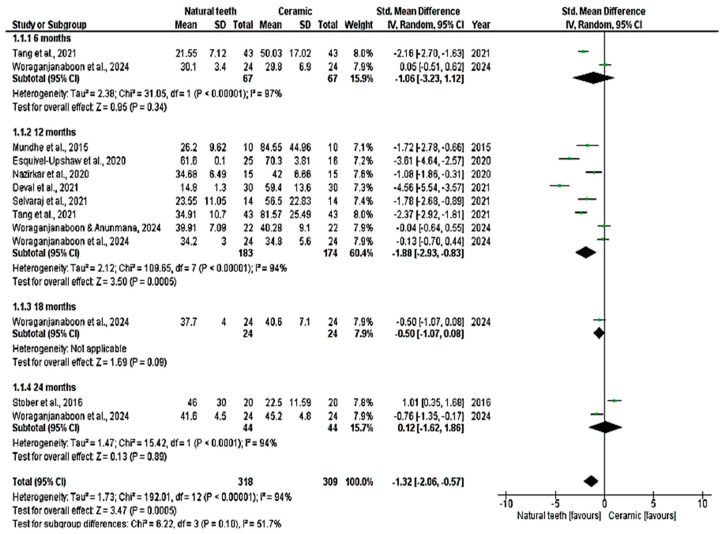
Meta-analysis of zirconia. Forest plot. IV—Inverse Variance [14,25,26,27,28,29,30,31].

**Figure 5:**
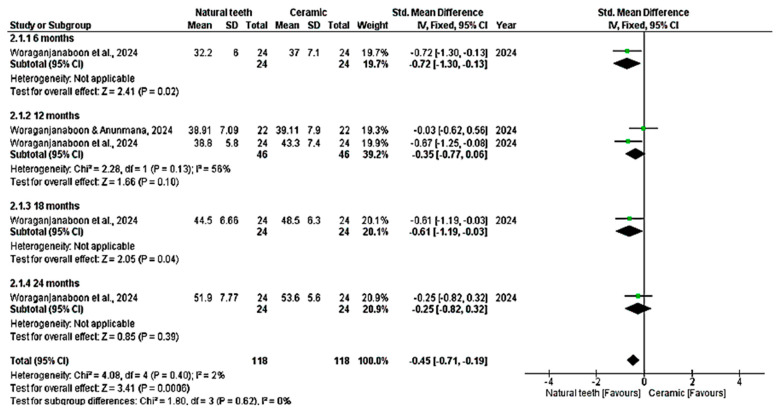
Meta-analysis of lithium disilicate. Forest plot. IV—Inverse Variance [31].

**Figure 6:**
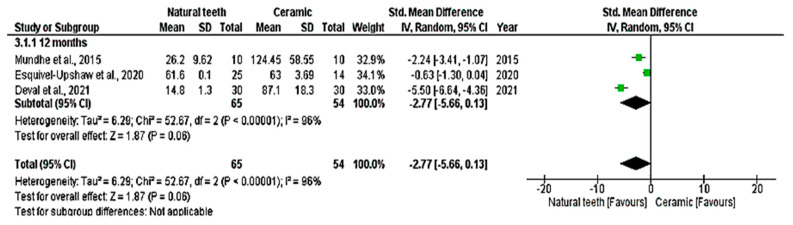
Meta-analysis of metal-ceramics (feldspathic). Forest plot. IV—Inverse Variance [14,25,28].

All nine studies were included in the quantitative analysis (meta-analysis), which evaluated enamel wear of the antagonist tooth in contact with crowns made of zirconia, lithium disilicate, and feldspathic metal-ceramic, compared with a control group of antagonist enamel against natural teeth. Vertical wear was measured in µm over 6, 12, 18, and 24 months. The meta-analysis showed significant enamel wear with zirconia ceramics (*p* < 0.05; MD: −1.32; 95% CI: −2.06 to −0.57; I^2^ = 94%, *p* < 0.001) and lithium disilicate (*p* < 0.05; MD: −0.45; 95% CI: −0.71 to −0.19; I^2^ = 2%, *p* = 0.40) over time (Figure 4 and Figure 5). Higher wear was observed with zirconia at 12 months (*p* > 0.05; MD: −1.83; 95% CI: −2.86 to −0.80; I^2^ = 94%, *p* < 0.001) and with lithium disilicate at 6 months (*p* = 0.02; MD: −0.72; 95% CI: −1.30 to −0.13) and 18 months (*p* = 0.04; MD: −0.61; 95% CI: −1.19 to 0.03). Feldspathic ceramic did not result in significant enamel wear (*p* = 0.06; MD: −2.77; 95% CI: −5.66 to 0.13; I^2^ = 96%, *p* < 0.001), as shown in Figure 6.

**Figure 7:**
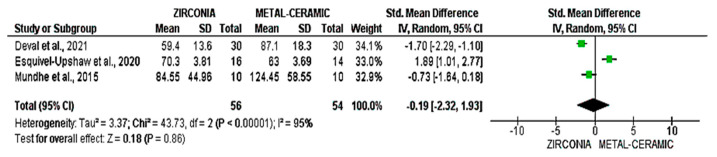
Meta-analysis of wear comparisons between metal-ceramic and zirconia [14,25,28].

A direct comparison of wear caused by metal-ceramic and zirconia was conducted in studies by Deval et al., 2021 [28], Esquivel-Upshaw et al., 2020 [14], and Mundhe et al., 2015 [25]. Deval et al., 2021 [28] observed that zirconia caused higher antagonist enamel wear, with a standardized mean difference (SMD) of −1.70 (95% CI: [−2.29; −1.10]). Esquivel-Upshaw et al., 2020 [14] found that metal-ceramic performed better, with an SMD of 1.89 (95% CI: [1.01; 2.77]), suggesting that metal-ceramic caused less wear compared to zirconia. Mundhe et al., 2015 [25] reported that the difference between the two materials was minimal, with an SMD of −0.73 (95% CI: [−1.64; 0.18]), indicating that wear was similar between the materials. The overall standardized mean difference across all three studies was −0.19 (95% CI: [−2.32; 1.93]). The *p*-value for the comparison was 0.86, indicating that the observed difference was not statistically significant (Figure 7).

### 3.5. Additional Analysis

The Kappa agreement test revealed inter-examiner agreement rates above 75% across all databases (PubMed/MEDLINE: 0.75; Embase: 0.78; Web of Science: 0.78; Cochrane Library: 1.0; and ProQuest: 1.0).

## 4. Discussion

This systematic review and meta-analysis evaluated which ceramic material causes the highest wear on opposing natural enamel in patients with single-unit crowns over time. The findings demonstrated significant enamel wear associated with zirconia and lithium disilicate crowns. In contrast, metal-ceramic crowns with feldspathic veneering showed no statistically significant difference in enamel wear compared with natural enamel-to-enamel contact (*p* = 0.06). This suggests that metal-ceramic crowns may wear opposing enamel, as in natural enamel antagonism. The null hypothesis was rejected, as statistically significant differences in enamel wear were found among the evaluated ceramic materials over time. This topic is clinically relevant because the restorative materials routinely used in daily practice may influence the long-term preservation of healthy antagonist enamel.

Tooth wear is a complex process that occurs over time and involves both chemical and mechanical factors. It is influenced by multiple variables, including the abrasive nature of food, masticatory behavior, parafunctional habits, neuromuscular forces, and the properties of the opposing ceramic materials, which vary in thickness, roughness, fracture toughness, and hardness [3,4,5]. The studies included in this systematic review and meta-analysis investigated ceramics with hardness values higher than that of enamel, which ranges from 3.14 to 3.72 GPa.

The age range of participants in the included studies was 18 to 73 years. This variation may influence enamel wear outcomes, as enamel hardness and structural integrity can change with age. Therefore, age-related variability should be considered when interpreting enamel wear results and planning future studies. Feldspathic ceramic, with a hardness closer to natural enamel (4.9 GPa), was the only material that did not exhibit significant wear over time, possibly due to this similarity. In contrast, zirconia has the highest hardness among ceramics, approximately 13 GPa [16].

The ceramic materials evaluated in this systematic review and meta-analysis caused more enamel wear on the opposing tooth than natural enamel, and this wear progressed over time [20,30,32]. Although zirconia showed significant enamel wear over time, the meta-analysis indicated that only the 12-month subgroup exhibited a statistically significant difference. In contrast, lithium disilicate showed substantial results in the 6- and 18-month subgroups. This supports the tribological concept of a ‘wear-in’ stage, which refers to the time required for the antagonist enamel to reach a stable wear pattern, an area that remains poorly understood in the literature [31,32].

Temporal analysis revealed that antagonist enamel wear may be more pronounced in the initial months of ceramic crown use and may stabilize thereafter. Zirconia wear peaked significantly at 12 months (MD = −1.83 µm; 95% CI: −2.86 to −0.80; *p* < 0.05), while lithium disilicate showed statistically significant differences at 6 months (*p* = 0.02) and 18 months (*p* = 0.04). This behavior may be linked to tribological softening, in which higher initial wear occurs until functional equilibrium between the surfaces is established. These findings underscore the importance of longitudinal follow-up to monitor wear stabilization and prevent excessive long-term enamel loss. This stabilization is explained by the increase in the area and number of wear facets, which reduce occlusal force per unit surface area and, consequently, vertical height loss, ultimately eliminating occlusal contact [4,16,32].

The cause of enamel wear around zirconia crowns remains controversial, with studies reporting both greater antagonist enamel wear and results comparable to natural enamel-to-enamel contact [8,9,10,32]. The meta-analysis, conducted to directly compare metal-ceramic and zirconia, based on the studies by Deval et al., 2021 [28], Esquivel-Upshaw et al., 2020 [14], and Mundhe et al., 2015 [25], shows variability in results regarding zirconia wear. Some studies indicate higher antagonist enamel wear (SMD of −1.70, 95% CI [−2.29; −1.10]), while others show no significant differences or even lower wear (SMD of −0.73, 95% CI [−1.64; 0.18]). This inconsistency may be due to the nature of enamel wear against zirconia, which tends to produce smooth wear with microscopic enamel chipping, small reticular cracks, and a corrosion-like structure, features that often require direct scanning and may need confirmation via scanning electron microscopy (SEM) [32]. The wear mechanism of lithium disilicate, however, is better understood because of its microstructure, which consists of crystalline particles that can create grooves in the antagonist enamel, leading to deformation, producing an abrasive surface, and encouraging further wear. These particles may also act as third-body abrasives [31,32].

The systematic review by Gou et al., (2019) [16] focused exclusively on zirconia and demonstrated that mechanically polished zirconia resulted in less antagonist tooth wear than glazed zirconia. This may be attributed to thinner, weaker glaze layers that, even without prior occlusal adjustment, deteriorate within the first few months of insertion, thereby increasing the risk of wear by exposing irregular surfaces. As reported by Gou et al., (2019) [16], mechanically polished zirconia exhibits wear characteristics similar to those of natural enamel. In contrast, glazed surfaces may degrade over time, increasing surface roughness and enamel abrasiveness. Despite this, the present review included studies by Stober et al., 2016 [26], Woraganjanaboon & Anunmana 2024 [31], and Woraganjanaboon et al., 2025 [32], all of which used glazed zirconia and still achieved favorable long-term outcomes. Nevertheless, to mitigate the adverse effects of rough ceramic surfaces on opposing enamel, restorations should be thoroughly mechanically polished after any occlusal adjustments performed during follow-up [16,32].

In most studies included in this systematic review and meta-analysis, Mundhe et al., 2015 [25], Esquivel-Upshaw et al., 2020 [14], Stober et al., 2016 [26], Selvaraj et al., 2021 [29], and Nazirkar et al., 2020 [27], enamel wear was analyzed using tridimensional laser optical scanners with impression replicas. This indirect method is currently considered the gold standard due to its high reproducibility. However, inadequate replication and alignment issues may introduce errors, resulting in variations of 0.61–0.7 [29]. In contrast, direct intraoral scanning eliminates the model fabrication step and reduces the risk of replica inaccuracies. This method was employed only by Tang et al., 2021 [30], Woraganjanaboon & Anunmana 2024 [31], and Woraganjanaboon et al., 2025 [32], and is considered more accurate, especially when combined with SEM analysis [31,32]. Therefore, due to methodological differences across studies, findings on enamel wear should always be interpreted with caution.

The literature demonstrates that the surface finish of ceramics plays a crucial role in their interaction with opposing enamel. The use of glaze versus sequential mechanical polishing on ceramics has distinct implications for antagonist enamel wear, and it is essential to understand the differences between these approaches. Although glaze is often used to create a smooth, glossy surface, it typically consists of a thin layer applied to the ceramic and may wear down more quickly over time. As noted by Stober et al., 2014 [26], glaze, being a superficial layer, can degrade with continuous use, exposing the underlying ceramic, which is often rougher. This phenomenon may increase the ceramic’s abrasiveness over time, potentially leading to greater wear of the opposing enamel [29].

In contrast, sequential mechanical polishing, as demonstrated by Selvaraj et al., 2021 [29] and Esquivel-Upshaw et al., 2020 [14], has proven to be a more effective option for reducing long-term wear. By polishing the ceramic with abrasives of varying grit sizes, surface roughness is controlled, resulting in a more durable surface that is less prone to degradation. The ceramic’s strength after polishing allows the restoration surface to maintain its integrity and avoid exposing rougher layers that could increase abrasiveness over time, as occurs with glaze [11,29]. The polished surface of the ceramic material, particularly when sequential polishing is employed, reduces roughness in a controlled manner, thereby minimizing abrasive wear on opposing teeth [29].

Therefore, the key difference between these two approaches lies in how they influence wear over time. While glaze initially provides an aesthetically pleasing, smooth surface, it may be more prone to wear due to its thinness and the eventual exposure of rougher ceramic layers as it wears down. Sequential mechanical polishing, on the other hand, using diamond abrasives and proper polishing techniques, offers a more sustainable solution for preserving antagonist enamel by maintaining ceramic surface strength and reducing abrasion on natural teeth over time [26,29].

Manually polished surfaces are less abrasive than glazed ceramics, as glaze layers may degrade over time, exposing more abrasive ceramic particles [14,32]. In the case of zirconia, studies suggest that mechanically polished surfaces with diamond paste have a wear coefficient similar to that of natural enamel. In contrast, glazed surfaces may become more abrasive over time. Such differences in surface treatment may partially explain the variability observed among the studies in this meta-analysis.

The high heterogeneity found in the meta-analysis for metal-ceramics (I^2^ = 97%) and zirconia (I^2^ = 94%) indicates substantial variability among the included studies. This heterogeneity may stem from methodological differences, including polishing techniques (glazing vs. manual polishing), wear measurement methods (intraoral scanning vs. impression replicas), and varying follow-up durations (6 to 24 months). Given this substantial methodological variability, the comparative results should be interpreted with caution, as some of the observed differences among ceramic materials may arise from heterogeneity in measurement techniques and finishing protocols rather than intrinsic material effects. Additionally, the chemical composition and mechanical properties of the ceramics, as well as patient-specific factors such as masticatory force and parafunctional habits, may contribute to the observed differences. Thus, future studies should aim for more standardized methodologies and better control of clinical variables to clarify the actual impact of these materials on enamel wear over time.

The findings of this meta-analysis emphasize the importance of selecting ceramic materials in daily clinical practice. Although the inter-material differences in enamel wear were minor, it remains reasonable for clinicians to prefer materials that provide an appropriate balance between mechanical strength and reduced antagonist wear. Moreover, our findings suggest that the commonly assumed relationship between higher material stiffness and increased enamel wear is not consistently supported across clinical studies. With larger sample sizes and future high-quality trials, these minor differences may become even less clinically meaningful. This nuance is essential for interpreting wear outcomes in a practical, patient-centered context.

For patients with a history of severe tooth wear or a high risk of enamel loss, metal-ceramic crowns may be the safest option due to their lower potential for antagonistic wear. Lithium disilicate crowns may be considered when higher mechanical strength is needed. Zirconia crowns may also be indicated in such cases, provided they are properly polished to minimize abrasiveness. Additionally, differences in participant age across studies may have contributed to variability in enamel wear, as aging alters enamel properties. Although the present review included studies with follow-up periods of up to 24 months, most investigations did not extend beyond 12 months. This predominance of short-term data limits the ability to evaluate the long-term behavior and stability of enamel wear against different ceramic materials, reinforcing the need for longer prospective studies.

## 5. Conclusions

Based on the results of this systematic review and meta-analysis, we conclude that ceramic materials generally produce greater enamel wear on opposing posterior natural teeth than enamel-to-enamel contact. Monolithic zirconia and lithium disilicate crowns caused higher antagonist wear than metal-ceramic crowns with feldspathic veneering, with the latter showing the most favorable behavior for preserving enamel integrity in posterior antagonists.

From a clinical standpoint, these findings suggest that metal-ceramic restorations remain a conservative and predictable choice for patients at higher risk of enamel loss in posterior teeth. Lithium disilicate may be selected when esthetics and moderate strength are required, while zirconia should be carefully polished and maintained to minimize abrasiveness against posterior natural antagonists. Clinicians should balance occlusal load, wear risk, material strength, and surface finishing protocols when selecting ceramic material for posterior crowns to reduce antagonist enamel wear. Longer follow-up studies are needed to confirm the long-term stability of these outcomes.

## Figures and Tables

**Table 1:** Search terms used in the electronic databases.

Database	Search Strategy	Search Terms
PubMed/MEDLINE	#1	((((((“fixed prosthodontics”)) OR (“fixed dental prosthesis”)) OR (“crown-tooth”)) OR (“tooth preparation”)) OR (“crown preparation”)) OR (“full crown preparation”)) OR (“full coverage restorations”)
	#2	(((((“ceramics”)) OR (“monolithic zirconia”) OR (“lithium disilicate”)) OR (“feldspathic”)) OR (“metal ceramic”)
	#3	(((“tooth wear”)) OR (“occlusal wear”)) OR (“enamel wear”)
	#4	#1 AND #2 AND #3
Web of Science	#1	((((((ALL=(“fixed prosthodontics”)) OR ALL=(“fixed dental prosthesis”)) OR ALL=(“crown-tooth”)) OR ALL=(“tooth preparation”)) OR ALL=(“crown preparation”)) OR ALL=(“full crown preparation”)) OR ALL=(“full coverage restorations”)
	#2	(((((“ceramics”)) OR (“monolithic zirconia”) OR (“lithium disilicate”)) OR (“feldspathic”)) OR (“metal ceramic”)
	#3	(((ALL= (“tooth wear”)) OR ALL=(“occlusal wear”)) OR ALL=(“enamel wear”)
	#4	#1 AND #2 AND #3
Embase	#1	((((((“fixed prosthodontics”)) OR (“fixed dental prosthesis”)) OR (“crown-tooth”)) OR (“tooth preparation”)) OR (“crown preparation”)) OR (“full crown preparation”)) OR (“full coverage restorations”)
	#2	(((((“ceramics”)) OR (“monolithic zirconia”) OR (“lithium disilicate”)) OR (“feldspathic”)) OR (“metal ceramic”)
	#3	(((“tooth wear”)) OR (“occlusal wear”)) OR (“enamel wear”)
	#4	#1 AND #2 AND #3
Cochrane	#1	((((((“fixed prosthodontics”)) OR (“fixed dental prosthesis”)) OR (“crown-tooth”)) OR (“tooth preparation”)) OR (“crown preparation”)) OR (“full crown preparation”)) OR (“full coverage restorations”)
	#2	(((((“ceramics”)) OR (“monolithic zirconia”) OR (“lithium disilicate”)) OR (“feldspathic”)) OR (“metal ceramic”)
	#3	(((“tooth wear”)) OR (“occlusal wear”)) OR (“enamel wear”)
	#4	#1 AND #2 AND #3
ProQuest	#1	(((((noft=(“fixed prosthodontics”)) OR (“fixed dental prosthesis”)) OR (“crown-tooth”)) OR (“tooth preparation”)) OR (“crown preparation”)) OR (“full crown preparation”)) OR (“full coverage restorations”)
	#2	((((noft=(“ceramics”)) OR (“monolithic zirconia”) OR (“lithium disilicate”)) OR (“feldspathic”)) OR (“metal ceramic”)
	#3	((noft=(“tooth wear”)) OR (“occlusal wear”)) OR (“enamel wear”)
	#4	#1 AND #2 AND #3

**Table 2:** Characteristics of the included studies.

Author and Year	Design	Patients	Age (Years)	Crown Position	Material of Ceramic	Follow-Up (Months)	Wear Results (Mean ± SD)		Conclusions
							Enamel × Enamel (µm)	Enamel × Ceramic (µm)	
Mundhe et al., 2015 [25]	RCT	10 NR	18–35	Molar and Premolar	Zr MC	12	26.2 ± 9.62	Zr 84.55 ± 44.96 Mc 124.45 ± 58.55	Zirconia crowns led to less wear of antagonist enamel than metal ceramic crowns, but more than natural enamel.
Stober et al., 2016 [26]	Prospective	10 M 10 F	21–73	Molar	Zr	6, 12, 24	6 months: NR 12 months: NR 24 months: 46 ± 30	6 months: NR 12 months: NR 24 months: 22.5 ± 11.59	Monolithic zirconia crowns generated more wear of opposed enamel than did natural teeth.
Esquivel-Upshaw et al., 2020 [14]	RCT	5 M 20 F	>21	Molar and Premolar	Zr MC	6 and 12	6 months: NR 12 months: 61.6 ± 0	Zr 6 months: NR 12 months: 70.3 ± 3.81 MC 6 months: NR 12 months: 63 ± 3.69	Monolithic zirconia exhibited comparable wear of enamel compared with metal-ceramic crowns and control enamel after one year.
Nazirkar et al., 2020 [27]	RCT	15 NR	20–40	Molar	Zr	12	34.68 ± 6.49	42 ± 6.66	Natural enamel wear is significantly more against zirconia crowns as compared to the natural antagonist.
Deval et al., 2021 [28]	RCT	30 NR	18–40	Molar	Zr MC	12	14.8 ± 1.3	Zr 59.4 ± 13.6 MC 87.1 ± 18.3	Wear of natural enamel opposing metal-ceramic and zirconia crowns was significantly higher than wear of natural enamel opposing natural teeth.
Selvaraj et al., 2021 [29]	RCT	14 NR	18–45	Molar and Premolar	Zr	12	23.55 ± 11.05	56.5 ± 22.83	Monolithic zirconia crowns cause more wear on the opposing natural enamel than on the natural enamel antagonists.
Tang et al., 2021 [30]	Prospective	22 M 21 F	±42.2	Molar and Premolar	Zr	6 and 12	6 months: 21.55 ± 7.12 12 months: 34.91 ± 10.7	6 months: 50.03 ± 17.02 12 months: 81.57 ± 25.49	The monolithic zirconia crown can cause more wear than natural teeth and will increase over time.
Woraganjanaboon & Anunmana 2024 [31]	Prospective	22 NR	18–66	Molar	Zr L	12	38.91 ± 7.09	Zr 40.28 ± 9.1 L 39.11 ± 7.90	This study found similar wear levels to enamel for both materials compared to natural teeth.
Woraganjanaboon et al., 2025 [32]	Prospective	24 NR	±37.13	Molar	Zr L	6, 12, 18 and 24	Zr 6 months: 30.1 ± 3.4 12 months: 34.2 ± 3.0 18 months: 37.7 ± 4.0 24 months: 41.6 ± 4.5 L 6 months: 33.2 ± 6.0 12 months: 39.8 ± 5.8 18 months: 44.5 ± 6.6 24 months: 51.9 ± 7.7	Zr 6 months: 29.8 ± 6.9 12 months: 34.8 ± 5.6 18 months: 40.6 ± 7.1 24 months: 45.2 ± 4.8 L 6 months: 37.0 ± 7.1 12 months: 43.3 ± 7.4 18 months: 48.5 ± 6.3 24 months: 53.6 ± 5.6	Lithium disilicate and 5Y-TZP crowns did not affect enamel wear more than enamel against enamel.

Note: The enamel wear measurement methods used in the included studies were as follows: Mundhe et al., 2015 [25], Stober et al., 2016 [26], Esquivel-Upshaw et al., 2020 [14], Nazirkar et al., 2020 [27], and Selvaraj et al., 2021 [29] used replica-based laser scanning. Tang et al., 2021 [30], Woraganjanaboon & Anunmana 2024 [31], and Woraganjanaboon et al., 2025 [32] used direct intraoral scanning (with scanning electron microscopy in the latter two studies). Deval et al., 2021 [28] did not specify the measurement method. Abbreviations: F, female; L, lithium disilicate; M, male; MC, metal-ceramic prosthesis (feldspathic); NR, not reported; Zr, zirconia.

**Table 3:** Polishing techniques of the included studies.

Author and Year	Polishing Technique	Reported Effect
Stober et al., 2014 [26]	Sequential mechanical polishing with diamond abrasives, followed by glaze application.	Polishing reduces the abrasiveness of zirconia, decreasing wear on opposing teeth.
Mundhe et al., 2015 [25]	Glazing for metal-ceramic ceramics, mechanical polishing for zirconia crowns (polishing protocol not specified).	Glazing and polishing are important for reducing enamel wear.
Esquivel-Upshaw et al., 2020 [14]	Silicone mechanical polishers (Denerica; Dental Corp., Toronto, ON, Canada) with coarse-to-fine grit for crown polishing. Final polishing with diamond paste (DirectDia Paste Diamond Polishing Paste; Shofu Dental Corp., Kyoto, Japan) applied with stiff bristle brush.	Polishing with low roughness is essential to reduce antagonist enamel wear.
Nazirkar et al., 2020 [27]	Sequential mechanical polishing with diamond abrasives, followed by glaze application.	Proper polishing reduces roughness and antagonist enamel wear.
Tang et al., 2021 [30]	Polishing with ceramic polishers impregnated with diamond abrasives (Dura-Polish DIA; Shofu Dental Corp., Kyoto, Japan).	Polishing reduces roughness and abrasiveness, minimizing enamel wear.
Deval et al., 2021 [28]	Not specified in the study.	Adequate polishing is crucial to minimize antagonist enamel wear.
Selvaraj et al., 2021 [29]	Sequential polishing with rotary diamond instruments (coarse, medium, fine, and superfine) for 20 to 30 s each.	Sequential polishing reduces zirconia roughness, minimizing antagonist enamel wear. Surfaces with controlled roughness cause less abrasion to natural teeth.
Woraganjanaboon & Anunmana 2024 [31]	Sequential polishing with diamond points: coarse, medium, and fine (ZilMaster; Shofu Dental Corp., Kyoto, Japan).	Sequential polishing is important for achieving low roughness, minimizing enamel wear.
Woraganjanaboon et al., 2025 [32]	Sequential polishing with diamond points: coarse, medium, and fine (ZilMaster; Shofu Dental Corp., Kyoto, Japan).	Sequential polishing helps reduce the abrasive impact on opposing teeth.

## Data Availability

Data sharing is not applicable to this article. No new data were created or analyzed in this study.

## Supplementary Materials

The following supporting information can be downloaded at: https://www.mdpi.com/article/10.3390/dj14010037/s1, Table S1: PRISMA 2020 Checklist.

## Abbreviations

The following abbreviations are used in this manuscript:

RCT	Randomized Clinical Trial
PRISMA	Preferred Reporting Items for Systematic Reviews and Meta-Analyses
PROSPERO	International Prospective Register of Systematic Reviews
PICO	Population, Intervention, Comparison, Outcome
IV	Inverse Variance
CI	Confidence Interval
SMD	Standardized Mean Difference
RoB 2.0	Risk of Bias 2.0
ROBINS-I	Risk Of Bias In Non-randomized Studies of Interventions
Zr	Zirconia
L	Lithium disilicate
MC	Metal-ceramic prosthesis
NR	Not reported
F	Female
M	Male
CAD-CAM	Computer-Aided Design and Computer-Aided Manufacturing
SEM	Scanning Electron Microscopy
Y-TZP	Yttria-stabilized tetragonal zirconia polycrystal
